# Efficacy of the Systemic Immune Inflammation Index in Malignant and Benign Parotid Neoplasms

**DOI:** 10.7759/cureus.31878

**Published:** 2022-11-25

**Authors:** Abdulkadir Sahin, Ayhan Kars, Korhan Kilic, Hasan Bera Ucar, Muhammed Sedat Sakat

**Affiliations:** 1 Otorhinolaryngology, Ataturk University, Faculty of Medicine, Erzurum, TUR; 2 Otorhinolaryngology, Erzincan Binali Yildirim University, Faculty of Medicine, Erzincan, TUR

**Keywords:** sii index, systemic immune-inflammation index, parotid mass, malignant tumor, inflammatory parameters, benign parotid tumor

## Abstract

Objective

Several studies have looked at systemic immune-inflammation index (SII) (neutrophil x platelet x lymphocyte) values, which have been shown to be useful in determining tumor aggressivity and prognosis, as well as predicting recurrence risk, particularly in cancer cases. The purpose of the current study was to determine SII values in patients with parotid masses and investigate their utility in distinguishing between malignant and benign parotid tumors.

Methods

This retrospective study included 237 adult patients-112 women and 125 men-who were followed up on and treated for parotid mass between 2015 and 2021. The SII values determined were compared between the groups.

Results

The difference between the two groups was statistically significant (p = 0.001). In addition, SII values were higher in malignant tumors with perineural and lymphovascular invasion compared to other malignant tumors, although the difference was not statistically significant.

Conclusions

Although SII values yielded significant results in differentiating malignant from benign parotid tumors, since no significant cut-off value was determined, we do not think that they represent an effective marker capable of being used to distinguish between these tumors in clinical practice.

## Introduction

Salivary gland tumors represent 3%-10% of all head and neck tumors, and 80% occur in the parotid gland [1,2]. Previous studies have shown that salivary gland cancers are the sixth most common head and neck cancers and constitute 0.3% of all malignancies. [3]. According to the 2017 WHO classification, there are 11 histologically benign types of parotid tumors and 24 malignant types, with each having unique characteristics [4]. The ability to determine the type of tumor preoperatively is of the utmost importance for accurate treatment planning but is generally not easy. Findings such as pain, tenderness, and facial paralysis are suggestive of malignancy in parotid gland masses. In addition, differentiating between malignant and benign masses is difficult due to the limited numbers of parotid carcinomas and to the findings in benign parotid masses being non-specific [4]. While ultrasonography (USG) and magnetic resonance imaging (MRI), used as the first step in preoperative evaluation, provide important information about the tumor location, studies involving functional MRI combined with advanced MRI techniques have yielded promising results in determining the tumor type with the highest sensitivity and specificity rates. Although USG-guided fine needle aspiration biopsy (FNAB) may be insufficient for determining the tumor type, it exhibits high accuracy rates for distinguishing benign from malignant in the hands of a good cytopathologist. Core needle biopsy is a method that can be used for diagnosis in situations in which FNAB is insufficient [5].

The cells that provide immune response development in the organism form as a result of hematopoietic stem cell differentiation. The immune response to microorganisms and tumor cells can protect the organism [2]. The relationship between cancer and inflammation is well known, and the cellular immune system is key to the inflammatory response [6]. Inflammation increases the risk of tumors, and inflammatory cells play an important role in neoplasm proliferation, invasion, and metastasis [7-9]. A number of recent studies [1] have investigated the systemic immune response in tumors. Peripheral cells associated with inflammation obtained from peripheral blood (neutrophils (N), lymphocytes (L), and platelets (P)) have been found to be related to tumor progression [9]. Recent studies have shown that inflammatory indices such as the N-L ratio (NLR), P-L ratio (PLR), and monocyte (M)-L ratio (MLR) may be associated with poor cancer prognosis [8]. In addition, the NLR has been described as capable of being used in differentiating malignant from benign salivary gland tumors [10]. It has also been suggested that the NLR can be used as a malignancy biomarker in salivary gland tumors [2]. The systemic immune-inflammation index (SII) has recently been shown to be capable of use as a prognostic marker in various malignancies [8]. The SII has also been described as a more objective and better marker than indices such as the NLR and PLR in showing the relationship between inflammation and immune response [9].

To the best of our knowledge, no previous study has examined the SII in parotid tumors. The purpose of this research was to investigate the usefulness of SII values in differentiating malignant from benign parotid tumors in patients presenting with parotid masses.

## Materials and methods

Approval for this retrospective study was granted by the Ataturk University Medical Faculty Clinical Research Ethical Committee (no. 2021/0152). The study included 237 patients who presented to the Ataturk University Medical Faculty Ear, Nose, and Throat Department with a parotid mass between 2015 and 2021 and were treated with superficial or total parotidectomy. All patients’ preoperative USG, MRI, and FNAB results were recorded.

Individuals with any hematological disease, cardiac diseases such as congestive heart failure or myocardial infarction, chronic kidney failure, inflammatory or autoimmune disease, acute or chronic infection, any other tumor or distant metastasis, diabetes mellitus, hypertension, or obstructive sleep apnea, or those on corticosteroid therapy were all excluded from the study. 

Clinical data were retrieved from patients' medical records. Demographic characteristics such as age and gender, previous complete blood count (CBC) results without receipt of any therapy, and postoperative histopathological findings were recorded. Blood specimens were collected from all patients in the morning one day before surgery. CBC measurements were performed on an automatic hematology analyzer (Sysmex XN-1000TM, Sysmex Europe GmbH, Japan). Based on postoperative pathology reports, patients were divided into two groups: malignant parotid tumor and benign parotid tumor. The patients with malignant parotid tumors were further subdivided into groups with and without perineural and lymphovascular invasion. P, N, and L results were used to calculate the SII (P x N/L). In the malignancy cases, SII values were compared between the malignant and benign groups, as well as between patients with and without perineural and lymphovascular invasion. 

IBM Statistical Package for the Social Sciences (SPSS) 20.0 was used for statistical analyses. The Shapiro-Wilk test was used for determining the distribution of data. As the distribution was normal, the Student's t-test was used for the comparison of data between the two groups. The chi-square test was used for the comparison of categorical data. A receiver operating characteristic (ROC) curve analysis was performed to establish a cut-off point. P<0.05 was regarded as significant for all tests.

## Results

The study included 237 patients ranging in age from seven to 91 years old, with 112 females and 125 males participating. A benign parotid mass was present in 185 (78.1%) patients and a malignant tumor in 52 (21.9%). The most common benign tumor was pleomorphic adenoma (125 patients, 67.5%), followed by Warthin tumor (32 patients, 17.2%). The most common malignant tumor was mucoepidermoid carcinoma (13 patients, 25%), followed by acinic cell carcinoma (9 patients, 17.3%), and ductal carcinoma (five patients, 9.6%). Benign neoplasia was observed in 87 female and 98 male patients, and malign neoplasia in 25 female and 27 male patients. No statistically significant gender difference was determined in terms of the benign-malignant ratio in parotid masses (p = 0.509). As expected, patients with malignant neoplasms were significantly older (p = 0.007). (Table 1)

SII values were 563.6±290.3 in benign neoplasias and 787.2±316.9 in malignant neoplasias and differed significantly between the two groups (p = 0.001) (Table I). ROC analysis revealed no significant SII cut-off value capable of being used in differentiating malignant and benign tumors. 

Perineural invasion was present in 11 of the patients with malignant neoplasia and lymphovascular invasion in 12. The mean SII value in the perineural invasion-positive patients was 812.54±414.2, while that in the patients without perineural invasion was 780.29±497.8. The difference between the two values was not statistically significant (p=0.871). Similarly, the SII value in the presence of lymphovascular invasion was 949.5±420.9, compared to 742.94±486.4 in the absence of lymphovascular invasion, and the difference between the two was again not significant (p = 0.294) (Table 2).

**Table 1:** Demographic properties All data are given as mean±SD SII: Systemic immune inflammatory index *: Statistically significant

	Benign (n=185)	Malignant (n=52)	p
Age	48.76±17.3	56.46±20.8	0.007*
Gender (M/F)	98/27	87/25	0.509
Histopathology (n)	Pleomorphic adenoma (125) Warthin (32) Others (28)	Mucoepidermoid carcinoma (13) Asinic cell carcinoma (9) Ductal carcinoma (5) Others (25)	
SII	563.6±290.3	787.2±316.9	0.001*

**Table 2:** SII of patients with lymphovascular and perineural invasion All data are given as mean±SD SII: Systemic immune inflammatory index

SII	Positive	Negative	p
Lymphovascular invasion (n=12)	949.5±420.9	742,94±486,4	0,294
Perineural invasion (n=11)	812.54±414.2	780,29±497,8	0,871

## Discussion

Malignant salivary gland tumors represent less than 5% of all head and neck cancers. Their clinicopathological spectra are broad, and they entail significant prognostic differences [2]. In addition, although swelling is the most common symptom in parotid gland tumors, the mass cannot be detected by palpation in 3%-20% of malignancies [4]. There are therefore still several difficulties in the diagnosis and management of parotid malignancies [2].

The relationship between cancer and inflammation has previously been investigated, and the effects of inflammatory cells on carcinogenesis have been reported [7]. Inflammation plays an important role in tumor development, invasion, and metastasis. Neutrophils, lymphocytes, mast cells, macrophages, and eosinophils all exhibit an effect in this process. Chemokines released by tumor cells, macrophage inflammatory protein 1, and interleukin (IL)-8 cause inflammatory cells to accumulate around the tumor, and tumors grow with the activation of these [1]. The neutrophils, platelets, and lymphocytes used to calculate SII values can affect the development of cancer in various ways. Neutrophils with important roles in tumor development, invasion, and progression also affect the production of angiogenic factors and cytokines [1,11]. Platelets and the coagulation system also facilitate the adhesion of tumor cells by releasing chemokines and cytokines and play an important role in cancer progression [6,7]. In contrast, lymphocytes exhibit their effect by preventing the proliferation and migration of tumor cells [7]. Lymphocytes play an important role in the elimination of cancer cells [11]. It may therefore be concluded that a high SII (NxP/L) is associated with more powerful inflammation, a weaker immune response, a disposition to malignancy, and a poor prognosis in patients.

Recent studies have shown a relationship between systemic inflammatory response levels and various tumors [2]. The SII, calculated from neutrophil, platelet, and lymphocyte counts at peripheral CBC, was recognized as a prognostic marker in 2014 and was used in several solid tumors [6]. High SII has been shown to be potentially associated with a poor prognosis [7]. To the best of our knowledge, the effect of the SII on salivary gland tumors, including parotid tumors, has not previously been investigated. One meta-analysis examining the effect of the SII on prognosis found that SII elevation was associated with poor prognosis in hepatocellular carcinomas, gastrointestinal tract cancers, urinary cancers, small-cell lung cancers, and acral melanomas. In those studies [9], no standard cut-off for SII was determined. In addition, a study of patients with hepatocellular carcinoma reported a poorer prognosis in patients with an SII >330 [12]. Another study found that high SII was associated with local recurrence in patients operated on due to early-stage squamous cell carcinoma (SCC) of the tongue [13]. An SII cut-off point of 715.739 was determined for patients with nasopharyngeal carcinoma in one study, which emphasized the value of SII in diagnosis and prognosis [14]. Another study described SII values as an independent prognostic factor in patients with laryngeal SCC, with a cut-off point of 517.64 [15]. No significant cut-off for SII could be determined in the present study. A high SII value has been linked to the histopathological presence of perineural and lymphovascular invasion in patients with tongue cancer [7]. A similar finding emerged in the present study, although this was not statistically significant.

A study of 209 patients with laryngeal lesions investigating NLR values in malignant, benign, and precancerous lesions reported significant elevations in malignant lesions [16]. A meta-analysis involving 6479 patients reported significant NLR elevations in cancers of the oral cavity, nasopharynx, hypopharynx, and larynx [17]. A study of 899 patients with laryngeal cancer observed statistically significant PLR elevation in advanced-stage cases [18]. Another study of 145 patients with parotid gland masses and 83 healthy controls reported higher NLR and PLR values in patients with malignant tumors than in those with benign tumors and also in the healthy population [2]. The SII investigated in the present study has been described as a better and more objective marker than indices such as NLR and PLR in showing the association between inflammation and immune response [8]. SII values in the present study were significantly higher in cases with malignant tumors.

Limitations

There are a number of limitations to this study, such as its retrospective and single-center nature. In addition, due to the low number of patients with perineural and lymphovascular invasion, the relationship between the SII and these histopathological findings, and thus the prognosis, could not be determined in a statistically significant manner. Further prospective studies with wider participation might be usefully performed on this subject.

## Conclusions

To the best of our knowledge, this is the first study to examine the value of the SII in patients with parotid tumors. Predicting the potential malignancy of a mass is critical for planning the appropriate surgical approach in patients with a parotid mass. Although SII values in this study yielded significant findings in terms of differentiating malignant from benign parotid tumors, since no significant cut-off point could be determined, we do not think that they are an effective marker for use in differentiating these tumors in clinical practice.
